# Sodium butyrate‐activated TRAF6‐TXNIP pathway affects A549 cells proliferation and migration

**DOI:** 10.1002/cam4.2564

**Published:** 2019-10-02

**Authors:** Xiaoqiang Xiao, Yanxuan Xu, Haoyu Chen

**Affiliations:** ^1^ The Joint Shantou International Eye Center of Shantou University and The Chinese University of Hong Kong Shantou China

**Keywords:** butyrate, NSCLC, TRAF6, TXNIP, ubiquitylation

## Abstract

TNF receptor‐associated factor 6 (TRAF6) promotes the development of human lung cancer through bridging RAS and NF‐kB pathways; on the other hand, thioredoxin‐interacting protein (TXNIP) suppresses the growth of tumors. However, the crosstalk between TRAF6 and TXNIP in non‐small cell lung cancer (NSCLC) is currently unclear. Here, we found that TXNIP expression induced by sodium butyrate (NaBu) was TRAF6‐dependent. Moreover, TXNIP interacted with TRAF6 via its PPxY motif. Polyubiquitylation analysis with wild‐type or mutant (Cysteine70 to Alanine) of TRAF6 further showed TRAF6 ubiquitylated TXNIP. NaBu reinforced the interaction of TRAF6/TXNIP as well as TXNIP’ polyubiquitylation. Moreover, treated with NaBu, the A549 cells with TRAF6/TXNIP double knockdown showed an enhanced protein expression of E‐cadherin comparing to cells with single gene or negative knockdown. The experimental results of transwell and nude mice xenograft showed that knocking down both TRAF6 and TXNIP in A549 cells affected its migration and proliferation compared to that of single knockdown or negative control cells. On the other hand, TXNIP localization was different depending on the cell types and fused‐tag (eg, FLAG or GFP). Our results revealed TRAF6 regulated the expression and polyubiquitylation of TXNIP in a NaBu‐dependent manner, alleviating tumorigenesis of TRAF6.

## Graphical abstract

TRAF6 interacted and uibquitylated TXNIP. Sodium butyrate promoted the interaction of TRAF6 and TXNIP. Traf6 mediated sodium butyrate induced‐TXNIP expression.

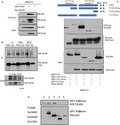

## 1 INTRODUCTION

Tumor necrosis factor receptor‐associated factor6 (TRAF6) is a member of the TRAF family, which mediates the signals from the Toll‐like receptor (TLR)/interleukin‐1 receptor (IL‐1R)^1^ and participates in the innate immune defense.^2^ Mechanistically, TRAF6 is an E3 ligase and catalyzes its substrate such as TAK1 (transforming growth factor‐β‐activated kinase 1) and Rac1^3^ to form a lysine‐63(K63)‐linked polyubiquitin chains.^4^ The ubiquitylated substrates in turn activate or conduct diverse biological functions including inflammation activation,^1^, ^4^ apoptosis induction,^3^ and other important cellular events.^5^ Recently, accumulating evidence points that TRAF6 promotes oncogenesis by increasing the HIF‐1a expression.^4^, ^6^, ^7^ Also, TRAF6 induces tumorgenesis of lung cancer through bridging the RAS and NF‐kB signaling.^4^ However, TRAF6 was also reported to be an antitumor gene in colon and liver cancer.^8^, ^9^

Thioredoxin‐interacting protein (TXNIP) is a member of *α*‐arrestin protein family and an endogenous inhibitor of thioredoxin (TRX) activity, leading to dissociation of TRX from apoptosis signal‐regulating kinase‐1, ER stress, phosphorylation of p38 and JNK, and subsequent cellular apoptosis.^10^ Hence, TXNIP has crucial biological function in cell proliferation and plays an important role in tumorgenesis.^11^, ^12^ Independent on its role in maintaining the redox balance in cells, TXNIP regulates glucose uptake via modulating the internalization and mRNA expression of GLUT1, a transporter of glucose.^13^ It was also reported that TXNIP activated inflammasome NLRP3, and thus promoted the maturation of pro‐caspase‐1 and proinflammation cytokine Pro‐IL‐1beta, in response to diverse stimuli.^12^ Structurally, TXNIP contains two characteristic arrestin‐like domains, PPxY motif which binds to the SH3 domain‐containing proteins or the WW domain‐containing proteins such as ITCH.^11^ Recently, the polyubiquitylation and phosphorylation of TXINP protein were uncovered. The ITCH‐mediated TXNIP polyubiquitylation promotes its degradation.^5^ On the other hand, the phosphorylation of TXINP on Ser308 is added by the AMP‐dependent protein kinase (AMPK), also leading to its degradation.^13^ The mRNA expression of TXNIP can be regulated by many factors including glucose, insulin, oxidative stress, inflammation, and fluid shear stress.^13^ Of those factors, suberoylanilidehydroxamic acid. A potent inhibitor of histone deacetlylases, induces TXNIP expression and arrests cell growth, differentiation and apoptosis.^14^

Previously, we found (NaBu, aninhibitor of histone deacetylases, induced TXNIP expression in A549 cells.^15^, ^16^ Moreover, NaBu suppressed the proliferation of A549 and promoted its death. NaBu is a short‐chain fatty acid^17^ and the fermentation products of dietary fibers metabolized by the intestinal microbiota.^18^ However, the inherent correlation of tumor suppressive gene TXNIP induction by NaBu with the oncogene TRAF6 in A549 is currently not well understood. In this study, we disclosed TRAF6 regulated the expression and polyubiquitylation of TXNIP in a NaBu‐dependent manner, decreasing the tumorigenesis of TRAF6.

## 2 MATERIALS AND METHODS

### 2.1 Cells culture, antibodies and reagents

In these experimental studies, human A549, HEK293T and Kyse150 cell lines (Chinese Academy of science, shanghai, China) were cultured in Dulbecco's modified Eagle's medium (DMEM; Gibco) supplemented with 10% fetal bovine serum, 1% glutamine, and 1% penicillin–streptomycin. All cells were maintained in a humidified atmosphere of 5% CO_2_ at 37°C. The antibodies of TXNIP, TRAF6, Caspase‐1/3, Bax, GAPDH, Tubulin, and beta‐Actin were purchased from Abcam Trading (Shanghai) Company (China). Anti‐Flag, anti‐HA antibodies, MG132 were obtained from Sigma. TRIzol and cDNA synthesis kit were from Invitrogen. The second antibodies were obtained from Biorad. Other antibodies were purchased from Cell Signaling Technology. Dual luciferase reporter assay kit was obtained from Promega. Lipofectamine® 3000 was purchased from Thermo Fisher Scientific. All other reagents were obtained from Shanghai Shenggong, China or Sigma. For reagent treatment, cells were loaded onto a culture dish 1 day before treatment with designated time points and concentrations.

### 2.2 Transfections and lentivirus infection

Cells were transfected with designated expression constructs via Lipofectamine3000 according to the provided protocol. For lentivirus infection, HEK293T cells were transfected with two virus package plasmids and the target plasmid, and the media were collected 48 hours posttransfection. Virus particles were purified with PEG8000 and kept in −80°C or directly used to infection. A549 cells (5 × 10^4^ cells/well) were seeded on 6‐well plates for 12 hours, infected by lentivirus (MOI = 20) with 5 μg/mL of polybrene for another 12 hours according to the manufacturer's protocol and then screened with puromycin (1 µg/mL). The cell lines, which can stably express the designated shRNAs or gene products, were then established through those methods.

### 2.3 Plasmids construction

For mammal cells TXNIP expression vectors construction, TXNIP sequence was cloned from A549 cells‐derived cDNA with primers (TXNIP GFP(wt) FP,GC AAGCTTATGGTGATGTTCAAGAAGATCAAGT; TXNIP GFP(wt) RP, GC GAATTCGCTCACTGCACATTGTTGTTGAGGAT), and inserted into pEGFP‐C3, 3×Flag‐tagged p‐FLAG‐CMV2 vector, respectively; or Primers: FP, ggatctatttccggtgaattc gccacc ATGGTGATGTTCAAGAAG; RP, agaactagtctcgaggaattc CTGCACATTGTTGTTGA) into pHB‐EF1‐MCS‐GFP. For construction of GFP‐merged TXNIP deletion mutants, the target sequence was cloned from the wild type of TXNIP with the same forward primer or reverse primer as well as the primer (GC GAATTC GCTGATCTGCTGCCAATTACCAGG) for TXNIP GFP(1‐281aa), or the primer (GCCTCGAGATGTTCGGCTTTGAG CTTCCTCAG) for TXNIP GFP (delete N‐100aa) or the primer (GCCTCGAG ATGGAGAATACATGTTCCCGAAT TGTG) for TXNIP GFP (delete N‐1‐200aa) and inserted into the same empty vector. Construction of GST‐Tagged TXNIP expression plasmids, TXNIP (WT) and deletion mutants were cloned from the pEGFP‐C3 TXNIP with the primers sets (CAGGAATTCATGGTGATGTTCAAGAAGATCAAG; GAACTCGAGTCACTGCACATTGTTGTTGAGGAT; for WT), (CAGGAATTCTTCGGCTTTGAGCTTCCTCAGG and for GST‐TXNIP (100‐400)), (GAACTCGAGTCATGATCTGCTGCCAATTACCAG) for GST‐TXNIP (1‐300) and inserted into pGEX‐5X‐1 vector. Sanger sequence was performed to confirm the sequence correction of constructed vectors. For the construction of TXNIP and TRAF6 shRNA expression plasmids (psi‐LVRH1GP), (Gaggtgtgtgaagttactc(ORF), Agacacgcttcttctggaa (ORF), Ttccaccgtcatttctaac (5UTR), and Ctctgacttcctaatgtag (3UTR) for ShTXNIP sequence；TTAGAGAGGTCACTTACTATT(3UTR), GCCACGGGAAATATGTAATAT (3UTR), CCCATCTGCTTGATGGCATTA (ORF) and CGAAGAGATAATGGATGCCAA (ORF) for shTRAF6) were bought from Shanghai Funeng company (Guangzhou China). The vectors could be packed by lentivirus. For TXNIP promoter activity assay, the promoter region of TXNIP was cloned from the genomic DNA of A549 cells and subcloned into pGL‐3Basic(our previously published paper). Flag‐TRAF6(C70A) mutant was kindly given by Dr Zongpin Xia.

### 2.4 Dual luciferase reporter assay

A549 cells expressing TRAF6 shRNA were seeded in 24‐well plates. Then, the cells were co‐transfected with either empty vector (control), or TXNIP promoter vector (pGL3‐basic‐TXNIP) together with internal plasmid‐expressing Renilla luciferase using Lipofectamine 3000 (Invitrogen). The transfected cells were treated or not treated with designated concentration of NaBu after a 12 hour transfection. And 36 hour later, the cells were lysed in passive lysis buffer (Promega), and luciferase activity was measured with dual luciferase assay kit (Promega, USA). Each group was analyzed in triplicate.

### 2.5 Co‐immunoprecipitation

TXNIP interaction with TRAF6 was tested by co‐immunoprecipitation (Co‐IP).

Total cell lysates (Nonidet P‐40 lysis buffer with protease inhibitor mixture (Roche)) for each sample were collected from three 10‐cm plates of HEK293Ts or A549s and incubated with 20 μL of prewashed Pierce™ Protein A/G Agarose beads (Thermo Scientific) at 4°C for 1 hour. The designated primary antibodies were then added to the precleared cell lysates and incubated at 4°C overnight. Then 40 μL of Protein A/G Agarose beads was added into each sample and incubated at 4°C for another 4 hours. Beads were centrifuged and washed with lysis buffer for at least 5 times. The beads‐specific‐binding protein compounds were collected and diluted with 40 μL of lysis Buffer (containing protease inhibitor) and were co‐IP products. All collected protein complexes were eluted with 10 μL of 5× loading buffer by boiling for 5 minutes and the elutes were subjected to SDS‐PAGE. For polyubiquitylation assay, the process is basically the same as the co‐IP except replacing the lysis buffer with RIPA buffer and one or two times washing with lysis buffer‐containing 6M urea.

### 2.6 GST‐binding assays

GST fusion proteins were induced in *Escherichia coli* BL21 cells by 0.25 mmol/L isopropyl‐*β*‐D‐thiogalactopyranoside (IPTG) for 12 hours or overnight. Cell pellets were resuspended in PBS containing 1% Triton X‐100, and then sonicated. The GST‐fused proteins were purified with glutathione‐Sepharose 4B beads (Sigma). For GST binding, 293 cell lysates expressing the target proteins were incubated with beads containing equal amounts of GST protein. Binding proceeded overnight with rotation at 4°C followed by five washes with lysis buffer. Bound proteins were released by boiling in gel‐loading sample buffer. All experiments were replicated at least once.

### 2.7 Nude mice xenograft for in vivo tumor growth assay

BALB/c nude mice were purchased from HFK Bioscience Company (Beijing, China) and bred under specific pathogen‐free conditions (Wenzhou medical university). All animals were used in accordance with institutional guidelines, and the current experiments were approved by the Use Committee for Animal Care of University. For subcutaneous inoculation, A549 shNC/shTXNIP and A549 sh*TXNIP/shTRAF6* cells (3 × 10^7^ cells) were, respectively, injected subcutaneously into the dorsal flank of each nude mouse (6 weeks old/8mice each group). When tumor grows to certain diameter of 150‐200 mm, remove the xenograft from sacrificed nude mice.

### 2.8 Statistical analysis

Statistical differences between two groups were assessed with the Student's *t* test. *P* < .05 was considered statistically significant. All results were expressed as mean ± SD from at least three independent experiments.

## 3 RESULTS

### 3.1 Endogenous and recombinant TXNIP showed different cellular localization

**Figure 1:**
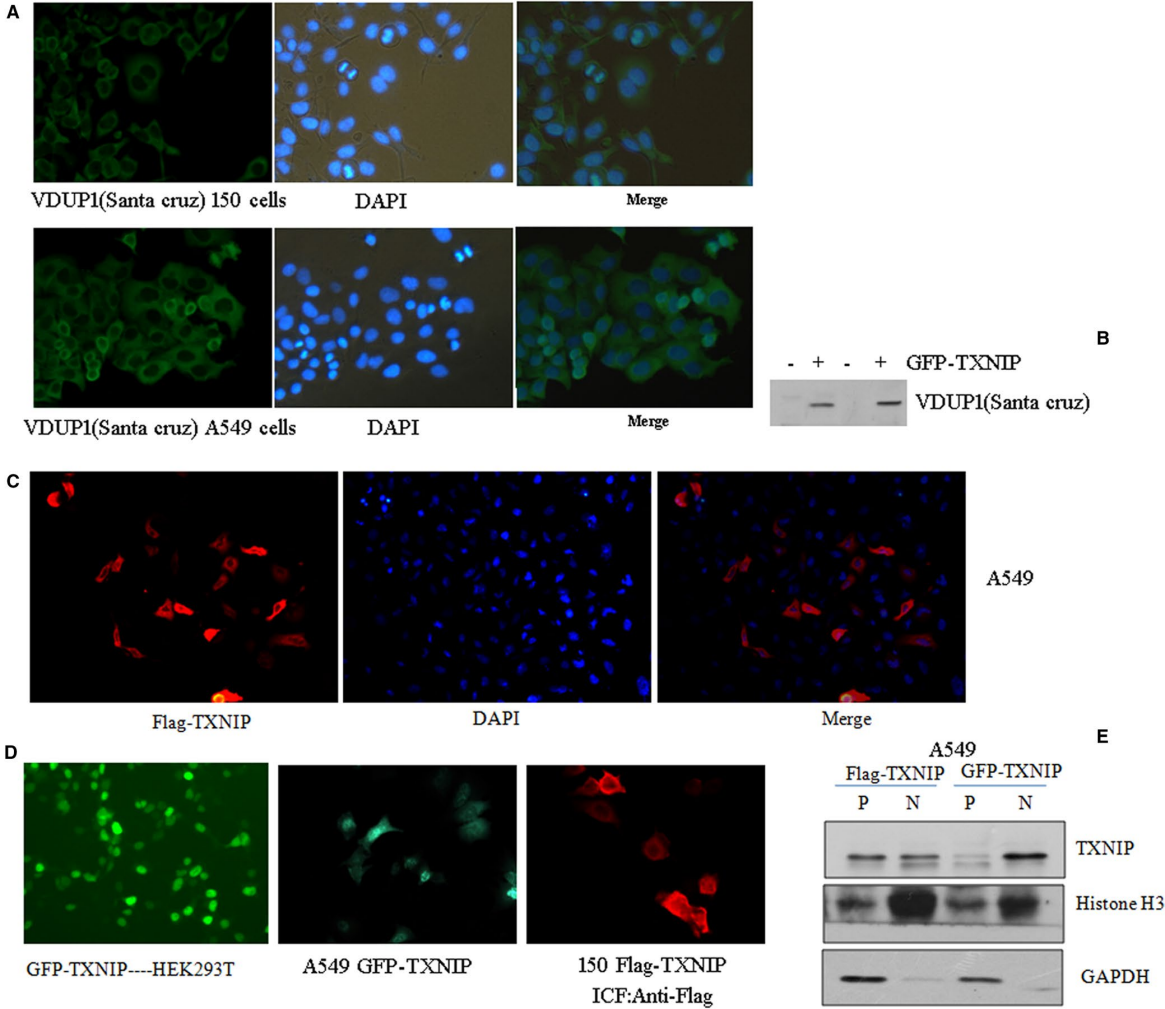
Localization of endogenous and recombinant TXNIP. A, Immunofluorescence staining was performed via TXNIP antibody from Santa cruz (VDUP1) in 150 (top) and A549 (low) cells. B, plasmids expressing GFP‐TXNIP protein were transfected into HEK293T cells. After 24 h transfection, cells were lysated and subjected to western blot using VDUP1 antibody. C, 3×Flag‐merged TXNIP were transfected into A549 and immunofluorescence staining for Flag‐TXNIP with anti‐Flag antibody. D, constructs expressing GFP‐TXNIP or Flag‐tagged TXNIP were transfected into HEK293T (Left), A549 (middle) for GFP‐TXIP and 150 (Left) for Flag tagged TXNP. E, A549 cells tranfected Flag‐TXNIP, or GFP‐TXNIP were used for cytoplasma protein and nucleus protein were extracted with NE‐PER Nuclear and Cytoplasmic Extraction Kit (Thermo scientific), and immunoblottings were then performed to test the TXNP expression with anti‐TXNIP antibody (Abcam). Histone H3 and GAPDH were used as a loading control for nucleus and cytoplasma protein, respectively. P, cytoplasma; N, nucleus

To understand the localization of TXNIP in cells, we firstly detected the localization of endogenous TXNIP in A549 and Kyse150 cells using anti‐TXNIP (VDUP1) antibody from Santa cruz via immunofluorescence staining. As shown in Figure 1A, TXNIP almost completely localized at the cytoplasm region in both 150 (up panel) and A549 (low panel), and the expression of this cytoplasmic TXNIP could not be induced by NaBu treatment (data not shown) when tested with this antibdoy. This antibody can also specifically detect the recombinant GFP‐merged human TXNIP protein overexpressed in HEK293T (Figure 1B), indicating the antibody work very well. On the other hand, our previously published data showed that endogenous TXNIP stained by antibody from Abcam could only be detected in the nucleus of A549 and TXNIP, which could be highly induced by NaBu treatment. To further confirm its cellular localization, we constructed GFP or Flag‐merged TXNP human recombinants. We transfected those two expression constructs into A549 cells, 150 cells, and HEK293T cells, we found that Flag‐merged TXNIP localized in both cytoplasm and nucleus in A549 (Figure 1C) and 150 (Figure 1D, left panel). TXNIP protein merged with GFP (GFP‐TXNIP) highly accumulated in the nucleus although a small amount of GFP‐TXNIP protein is present in the cytoplasm of both HEK293T (Figure 1D right panel) and A549 (Figure 1D middle panel). The localization of Flag‐/GFP‐merged TXNIP was also confirmed by immunoblotting after separating the protein component of cytoplasma and nucleus (Figure 1E). We also observed that a spontaneous mutation cT140C, pL47P in TXNIP cloned from A549 cell, which localizes at both cytoplasm and nucleus in GFP‐fused manner (data not shown). Those results indicate the localization of TXNIP protein in cells is complex.

### 3.2 TRAF6‐mediated NaBu‐induced TXNIP expression in A549

**Figure 2:**
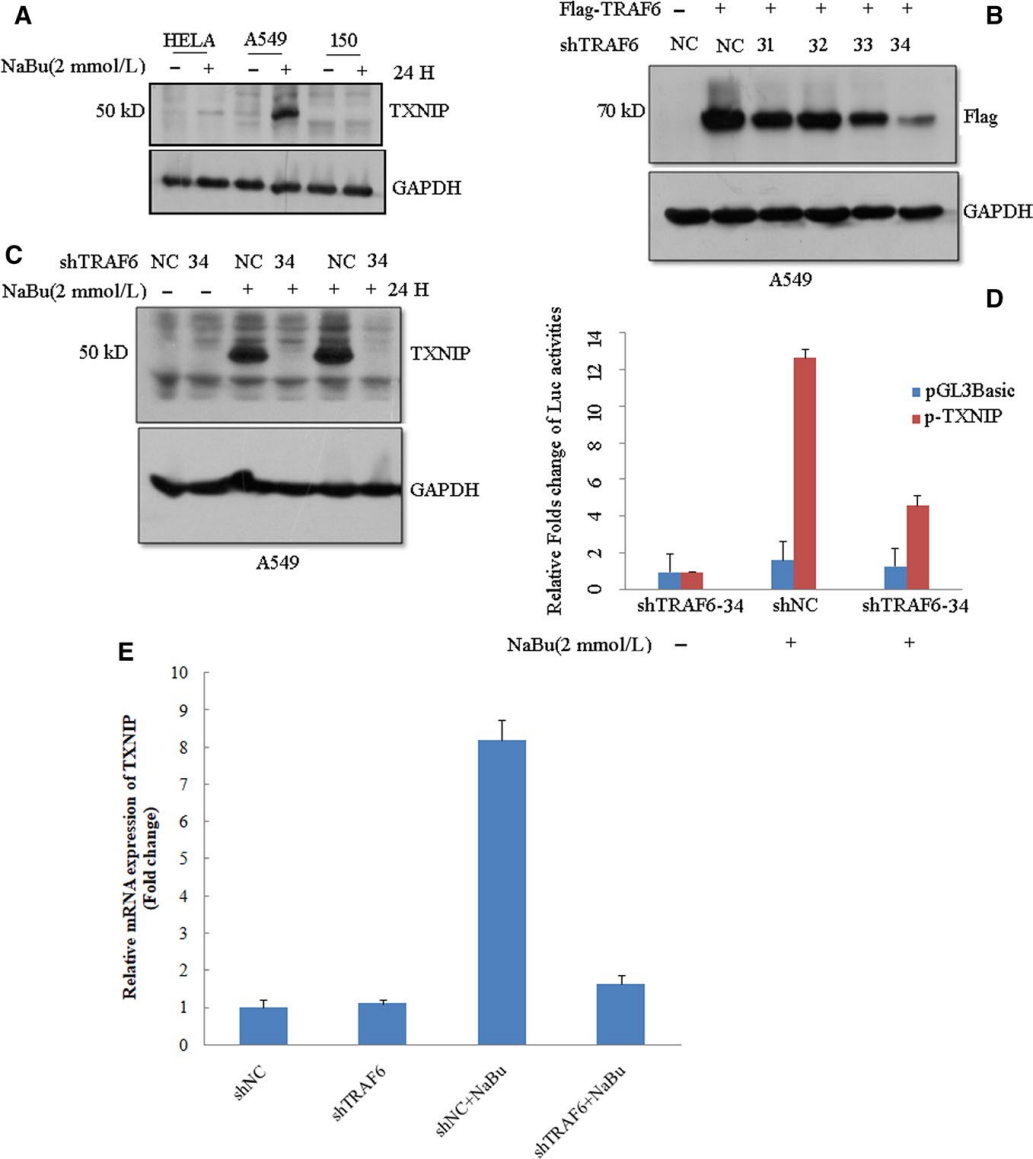
TRAF6 knockdown decreased NaBu‐induced TXNIP expression in A549 cells. A, Hela, A549 and Kyse150 cell lines were incubated with 2 mmol/L NaBu for 24 h, immunoblotting was used to detect the expression of TXNIP with anti‐TXNIP antibody from Abcam. B, HEK293T cells were co‐transfected with Flag‐TRAF6 and TRAF6 shRNA (sh31, sh32, sh33 and sh34) expression vectors. After 48 h, cells were lysated and subjected to western blot with anti‐Flag antibody. C, A549 cells stably expressing scramble shRNA (NC) or TRAF6 shRNA (sh34) were treated with or without 2 mmol/L NaBu for 24 hours. Lysates were subjected to western blot with anti‐TXINP (abcam) to detect the TXNIP expression. D, Plasmids containing TXNIP promoter sequence (p‐TXNIP) or corresponding empty vectors (pGL‐3Basic) together with the internal control vector renilla luciferase were co‐transfected into TRAF6 stably knockdown or scramble shRNA (NC) A549 cell line. Here 12 h after transfection, cells were treated with or without 2 mmol/L NaBu for another 36 h. Then, cells were lysated and analyzed with dual reporter luciferase assay kit.E, mRNA expression of TXNIP in A549 cells stably expressing the TRAF6 shRNA(shTRAF6) or negative control shRNA(shNC) were treated with 5 mmol/L sodium butyrate for 24 hours. Cells were then were lysated with Trizol and used for total RNA extraction. Experiments were performed in triplicates. Results were shown as the mean values (±SD)

Previously, we found NaBu could induce a large number of genes expression in A549 cells. Of those genes, TXNIP is one of the highly induced genes. Here, we further assessed the TXNIP expression in different cell lines, including Hela, A549, and Kyse150 cell lines. Among these three cell lines, A549 showed the highest TXNP protein expression after NaBu treatment, indicating that TXNIP expression are more sensitive to NaBu stimuli in A549 cells (Figure 2A). To further understand the molecular mechanism, we stably knocked down the expression of TRAF6 in A549 with constructs expressing shRNA of TRAF6 (sh31‐34). The knockdown efficiency was confirmed by immunoblotting. We revealed that the construct which expresses sh34 has the highest knockdown efficiency among the four shRNAs constructs (Figure 2B). A549 cells expressing TRAF6 sh34 or its scramble shNC were then treated with or without 2 mmol/L NaBu for 24 hours, the expression of TXNIP protein was detected by immunoblotting. Unexpectedly, TXNIP expression was almost abolished in A549 cells expressing sh34 in comparison with the shNC (negative control) after NaBu treatment for 24 hours (Figure 2C). We also detected the mRNA expression of TXNIP under the same treatment. As expected, mRNA expression of TXNIP was largely declined in TRAF6 knockdown cells (Figure 2E). The result prompted us to further analyze the promoter activity of *TXNIP* gene. We cloned the promoter region and inserted into pGL‐3 Basic vector, which resulted in a plasmid named pTXNIP. After transfecting pTXNIP or its empty vector (pGL3basic) together with the internal vector expressing renila luciferase into A549 cells, which stably express sh34 or shNC, for 12 hours, NaBu was then adminstrated into the culture medium and treated for another 24 hours. The promoter activities were detected by dual reporter luciferase kit (Promega). The luciferase activity of pTXNIP significantly decreased in cells with TRAF6 sh34 expression as compared with the negative control (shNC) (Figure 2D). This result was consistent with the trend of TXNIP protein expression. Together, the above results indicate that TRAF6 mediates NaBu‐induced TXNIP expression in A549 cells.

### 3.3 TRAF6 interacts with TXNIP

**Figure 3:**
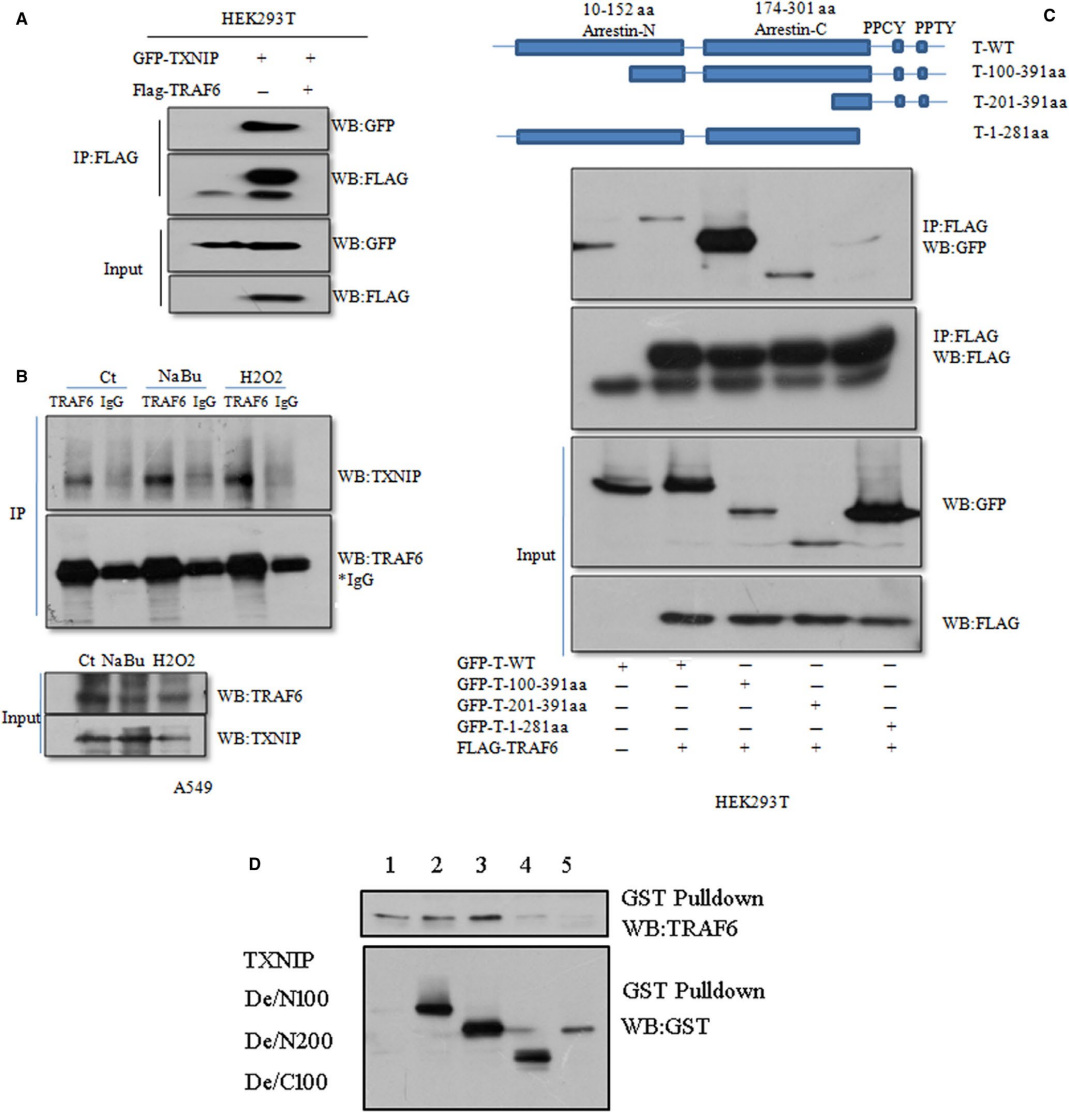
TXNIP interacts with TRAF6 via PPxY motif and NaBu strengthens endogenous TXNIP/TRAF6 interaction. A, GFP‐TXNIP were co‐transfected with Flag‐TRAF6 or its empty vector into HEK293T cells for 30 h, cells then lysated with 1 × NP‐40 lysis buffer containing complete protease inhibitors and anti‐GFP antibody was used for the immunoprecipitation (IP) analysis. The pull‐down protein and whole lysates were subjected to western blot with Anti‐GFP and anti‐Flag antibody. B the endogenous interaction was tested with A549 cells treated with a designated concentration of H2O, NaBu, and H_2_O_2_ for 24 h, then using the anti‐TRAF6 or IgG antibody to immunoprecipitated endogeneous TXNP. Anti‐TXNIP (Abcam) and anti‐TRAF6(Abcam) were used subsequent to western blot. C, TXNIP deletion mutants merged with GFP were co‐transfected with or without Flag‐TRAF6 into HEK293T cells, respectively, and cell culture continued for 30 h. Cells were lysated and used for IP assay with anti‐Flag antibody. GFP‐T‐WT: TXNIP wild type, GFP‐100^391aa: TXNIP N‐terminus 100 amino acid residues were deleted. GFP‐201^391aa: TXNIP N‐terminus 200 amino acid residues were deleted. GFP‐1^281 aa: C‐terminus 100 amino acid residues were deleted, including the PPxY domain of TXNIP. D, Bacterially expressed GST fusion proteins of wild‐type (WT), deletion mutant (100‐391aa), deletion mutant (200‐391aa), and deletion mutant(1‐300aa) of TXNIP were bound to glutathione‐Sepharose beads as indicated and incubated with lysates of HEK293T cells transfected with a Flag‐TRAF6 expression construct. Bound Flag‐TRAF6(Upper panel), GST‐TXNIP (bottom panel) were subjected to western blot with anti‐TRAF6 and anti‐GST antibodies, respectively

As an important tumor repressive gene, TXNIP expression induced by NaBu is TRAF6‐dependent. TRAF6 is also a well‐known E3 ligase of polyubiquitylation. Therefore, we further investigated the potential interaction between TXNIP and TRAF6 via co‐immunoprecipitation (CoIP). GFP‐TXNIP was transfected into HEK293T with Flag‐TRAF6 or with empty vector for 30 hours and lysated with 1 × NP40 buffer containing the complete protease inhibitors. Co‐IP was performed with anti‐Flag antibody. The results of Co‐IP showed a strong GFP band at the size about to 70 kD in GFP‐TXINP and Flag‐TRAF6 co‐expression group but not in GFP‐TXINP single expression group (Figure 3A). Both constructs successfully expressed the target protein as shown by the input in Figure3A. To further assess endogenous interaction of TXNIP and TRAF6 proteins in response to NaBu or H_2_O_2_ stimuli, we then used anti‐TRAF6 and IgG antibody to immunoprecipitate endogenous TXNIP protein. The TXNIP protein band was easily observed in TRAF6, but not in IgG. (Figure 2B). Furthermore, we found that cells with NaBu or H_2_O_2_ treatment enhanced the concentration of the pull‐down TXNIP protein, suggesting a strengthened interaction of TXNIP/TRAF6 protein (Figure 2B). To elucidate the domain of TXNIP protein interacting with Flag‐TRAF6, we constructed TXNIP deletion mutants. And then co‐expressed the mutants with Flag‐TRAF6 in HEK293T. Anti‐Flag antibody (sigma) was used for the co‐immunoprecipitation experiments. We found that Flag antibody could not pull down the TXNIP mutant with PPxY motif deletion although it highly expressed (Figure 3C). Interestingly, the mutant with 100 amino acid residues deletion in N‐terminus was easily pulled down as shown in a thick blotting band (Figure 3C). Those results suggested PPxY motif was involved in TXNIP/TRAF6 interaction. The interaction between TXNIP and TRAF6 was also observed in the GST pull‐down experiment (Figure 3D).

### 3.4 TRAF6 promotes TXNIP polyubiquitylation

**Figure 4:**
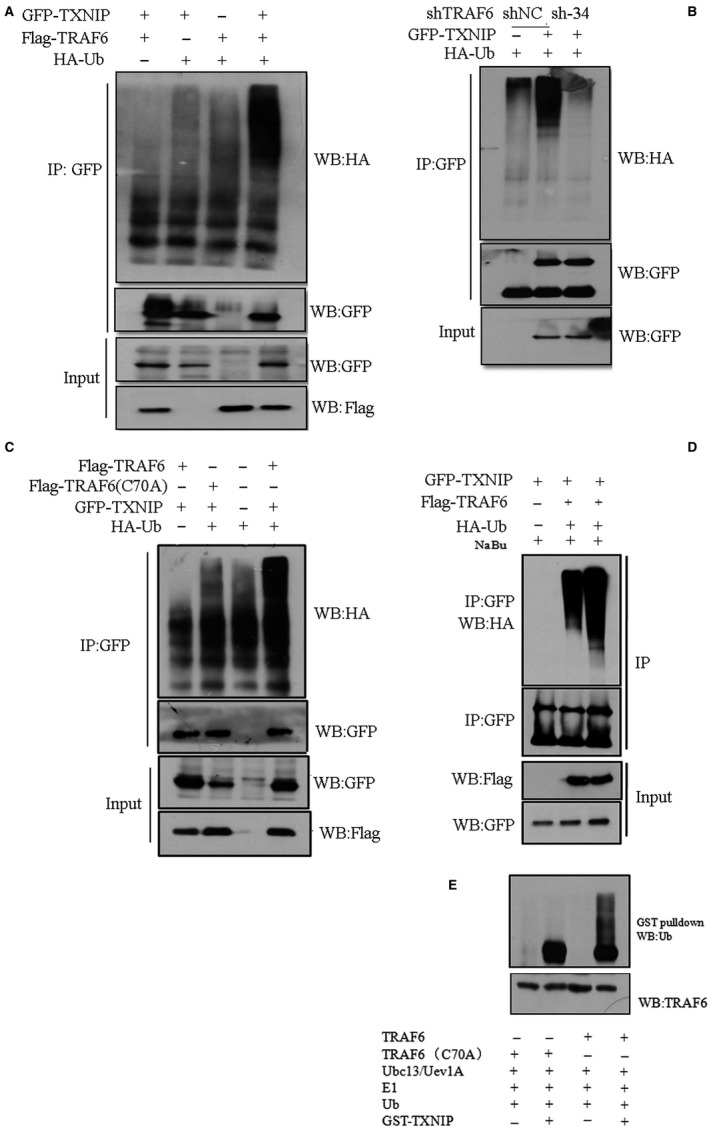
TRAF6 polyubiquitylated TXNIP. A, Expression construct of GFP‐TXNIP was co‐transfected with Flag‐TRAF6 or corresponding empty vector and/or HA‐Ub/ empty vector into HEK293T cells. After 36 h transfection, cells were lysated with 1 × RIPA buffer containing complete inhibitors and used for IP assay with anti‐GFP antibody. During the washing step, we added 6 mol/L urea into the washing buffer at the second wash step. Thereafter, immunoblotting assay was performed with anti‐HA (IP)/anti‐Flag (Lysates)/anti‐GFP(IP/Lysates) antibody. B, A549 cells stably expressing TRAF6 shRNA (sh‐34) or scramble RNA (shNC) were co‐transfected with GFP‐TXNIP or its corresponding empty vector together with vector expressing HA‐Ub. IP was performed with anti‐GFP antibody post‐48 h transfection. The eluted IP protein was subjected to western blot with Anti‐HA antibody or anti‐GFP antibody. C, GFP‐TXNIP construct was co‐transfected with Flag‐TRAF6 (wild type) or Flag‐TRAF6 (C70A mutant) constructs together HA‐Ub construct into HEK293T cells. The IP process is the same as A, or B. anti‐HA antibody was used for the detection of polyubiqutylation of TXNIP. D, the plasmids transfected into HEK293T cells was the same as in A. After transfection, cells were treated with 2 mmol/L NaBu for 24 h, and IP was performed as A using GFP antibody. Anti‐HA antibody was used for the immunoblotting detection. E, Bacterially expressed and purified GST‐TXNIP proteins were incubated with Flag‐TRAF6 or Flag‐TRAF6/C70A mutant in the presence of E1, E2 (Ubc13/Uev1A), and ubiquitin (Ub). Following the ubiquitination reaction, the TXNIP‐ubiquitin conjugates were detected by GST pull‐down and immunoblotted with anti‐Ub, anti‐GST and anti‐TRAF6, respectively

In light of the physical interaction between TRAF6 and TXNIP, we thus determined the polyubiquitiylation of TXNIP in the context of TRAF6 overexpression or knockdown. We found that TRAF6 overexpression in HEK293T cells could slightly increase the polyubiquitilation of TXNIP (Figure 4A). On the other hand, knocking down TRAF6 expression with sh34 in A549 cells, reduced the level of TXNIP polyubiqutylation (Figure 4B). It was reported that Cysteine (C70) residue in TRAF6 is critical for its E3 ligase activity. Therefore, we mutated cysteine(C) to alanine (A) named Flag TRAF6 C70A. Then we compared the level of TXINP polyubiquitylation in HEK293T cells expressing wild‐type TRAF6 (Flag‐TRAF6) and C70A mutated TRAF6. After 30 hours transfection, cells were lysed and used for IP. As expected, we found that the level of TXNIP polyubiquitylation declined in Flag‐TRAF6 (C70A) comparing to that in Flag‐TRAF6 via anti‐HA antibody (Figure 4C). Next, we investigated the effect of NaBu on the TRAF6‐mediated TXNIP polyubiquitylation. A549 cells expressing either GFP‐TXNIP/Flag‐TRAF6/HA‐Ub or only GFP‐TXNIP were treated with NaBu for 24 hours. IP was performed with GFP antibody. The subsequent results showed that NaBu treatment could slightly increase the level of polyubiquitylation of TXNIP (Figure 4D). Moreover, We observed a TRAF6‐mediated TXNIP polyubiquitylation by in vitro ubiquitylation reaction system (Figure 4E).

### 3.5 TRAF6 affects TXNIP stability and TXNIP promotes pro‐caspase3, but not pro‐caspase1 activation in a NABU‐dependent manner

**Figure 5:**
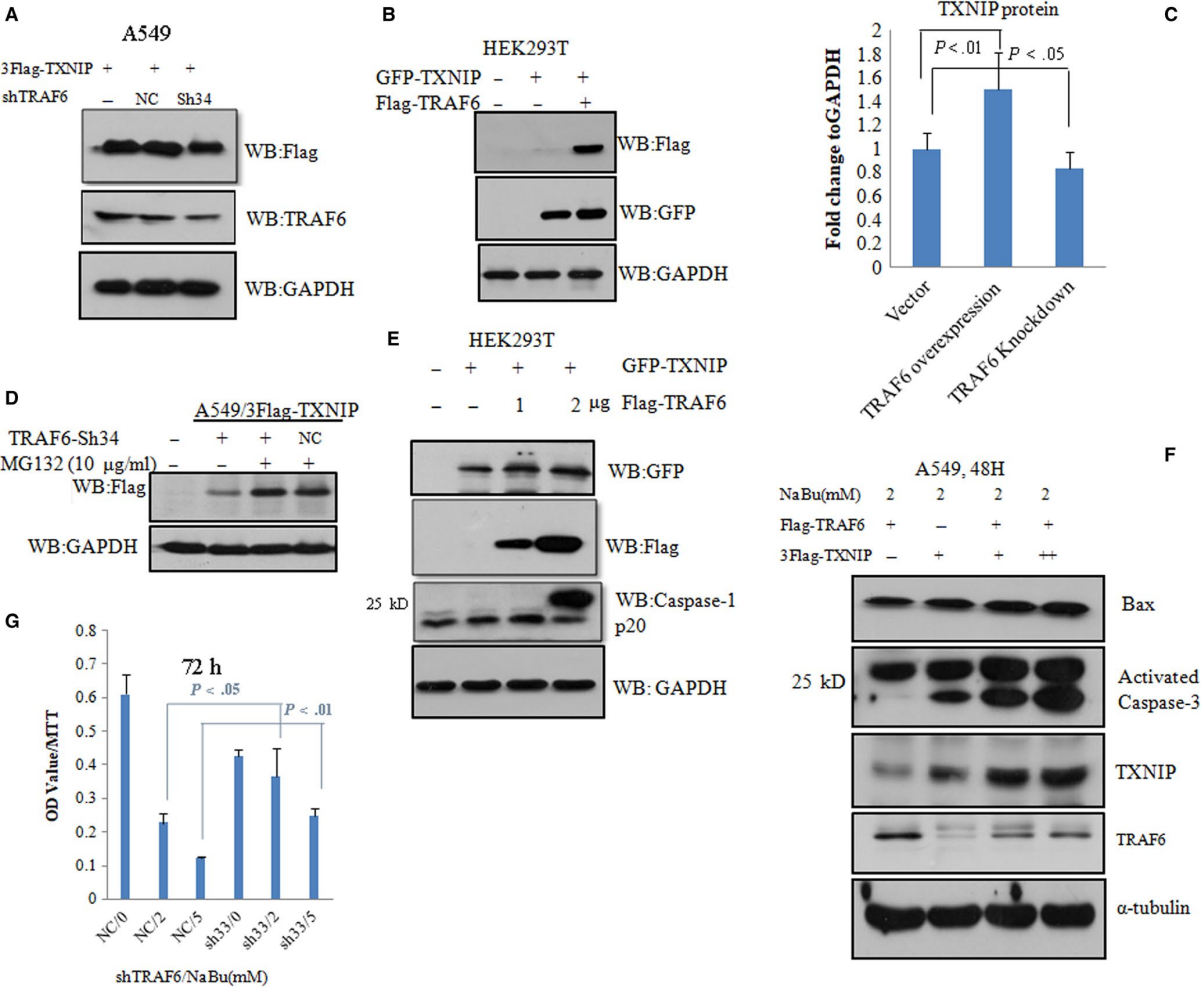
TRAF6 slightly enhanced TXNIP stability and TXNIP‐mediated pro‐caspase‐3 but not pro‐caspase‐1 activation. A, A549 cells stably expressing sh‐scramble RNA (NC) and TRAF6 shRNA(sh34), or wild type A549 were transfected with an expression construct for 3 Flag‐TXNIP. After 48 h, cells were lysated and subjected to western blot with anti‐Flag and anti‐TRAF6 antibody, respectively. B, expression constructs of GFP‐TXNIP was co‐transfected with Flag‐TRAF6 or its empty vector in HEK293T cells. The level of TXNP and TRAF6 protein levels was detected by immunoblotting with anti‐GFP and anti‐Flag antibody, respectively. C, The quantification of immunoblot for A, B is shown in C. The mean values (±SD) of three independent experiments are shown. D, A549 cells expressing sh‐scramble RNA(shNC) or TRAF6 shRNA(sh34) were infected with lentivirus construct expressing 3Flag‐TXNIP for 18 h, then treated with MG132 to another 12 h, Flag‐TXNIP protein was detected by immunoblotting with anti‐Flag antibody. E, expression construct for GFP‐TXNIP was co‐transfected with different amount of expression constructs for Flag‐TRAF6(1 ,2 µg) or corresponding empty vector into HEK293T cells. Cell lysates were subjected to western blot with anti‐caspase‐1(activated), anti‐GFP and anti‐Flag. F, different concentration plasmids of 3Flag‐TXNIP and Flag‐TRAF6 were co‐transfected into A549 for 24 h, then treated with 2 mmol/L NaBu for 48 h, Cell lysates were subjected to western blot with anti‐capsase‐3 (activated), anti‐Bax, anti‐TRAF6 and anti‐TXNIP antibodies. G, MTT results for triplicates. A549 cells with TRAF6 knockdown or not were treated with 0, 2, 5 mmol/L respective, for 72 h and then subjected to MTT analysis. All samples were detected in triplicates. In all above experiments, GAPDH or Tubulin as a loading control. Student‐*t* test was used for the *P* value assay

To understand the biological roles of TXNIP ubiqutylation driven by TRAF6, we primarily analyzed the stability of TXNIP protein in cells with TRAF6 either overexpression or knockdown. As expected, TRAF6 overexpression in HEK293T enhanced the amount of TXNIP protein (Figure 5B,C); however, knocking down TRAF6 could slightly but significantly decrease the TXNIP protein level(Figure 5A,C). MG132 is an inhibitor of proteasome and prevents polyubiquitylated‐protein degradation. Therefore, A549 cells stably expressing TRAF6 sh34 or scramble shNC were transfected with Flag‐TXNIP, and then those cells were treated with 10 μmol/L MG132 for 12 and 24 hours, respectively. Then using immunoblotting, we checked the expression of TXNIP protein. The result showed that the expression level of TXNIP protein enhanced in comparison with the nontreated control (Figure 5D). The gradient increase in TRAF6 protein in HEK293T cells expressing GFP‐TXNIP slightly inhibited the activation of caspase‐1 (Figure 5E). Surprisingly, NaBu‐treated A549 cells promoted caspase‐3 activation and Bax expression depending on the level of TRAF6 and TXNIP protein (Figure 5F). Finally, we measured the proliferation of A549 cells expressing TRAF6 sh34 or scramble shNC treated with NaBu via 3‐(4,5‐dimethyl‐2‐thiazolyl)‐2,5‐diphenyl‐2‐H‐tetrazolium bromide (MTT). We found the knockdown of TRAF6 expression showed more resistantance to NaBu treatment in comparison to negative control (NC) (Figure 5G).

### 3.6 NaBu‐dependent TRAF6‐TXNIP signal affects the proliferation and migration of A549 cells

**Figure 6:**
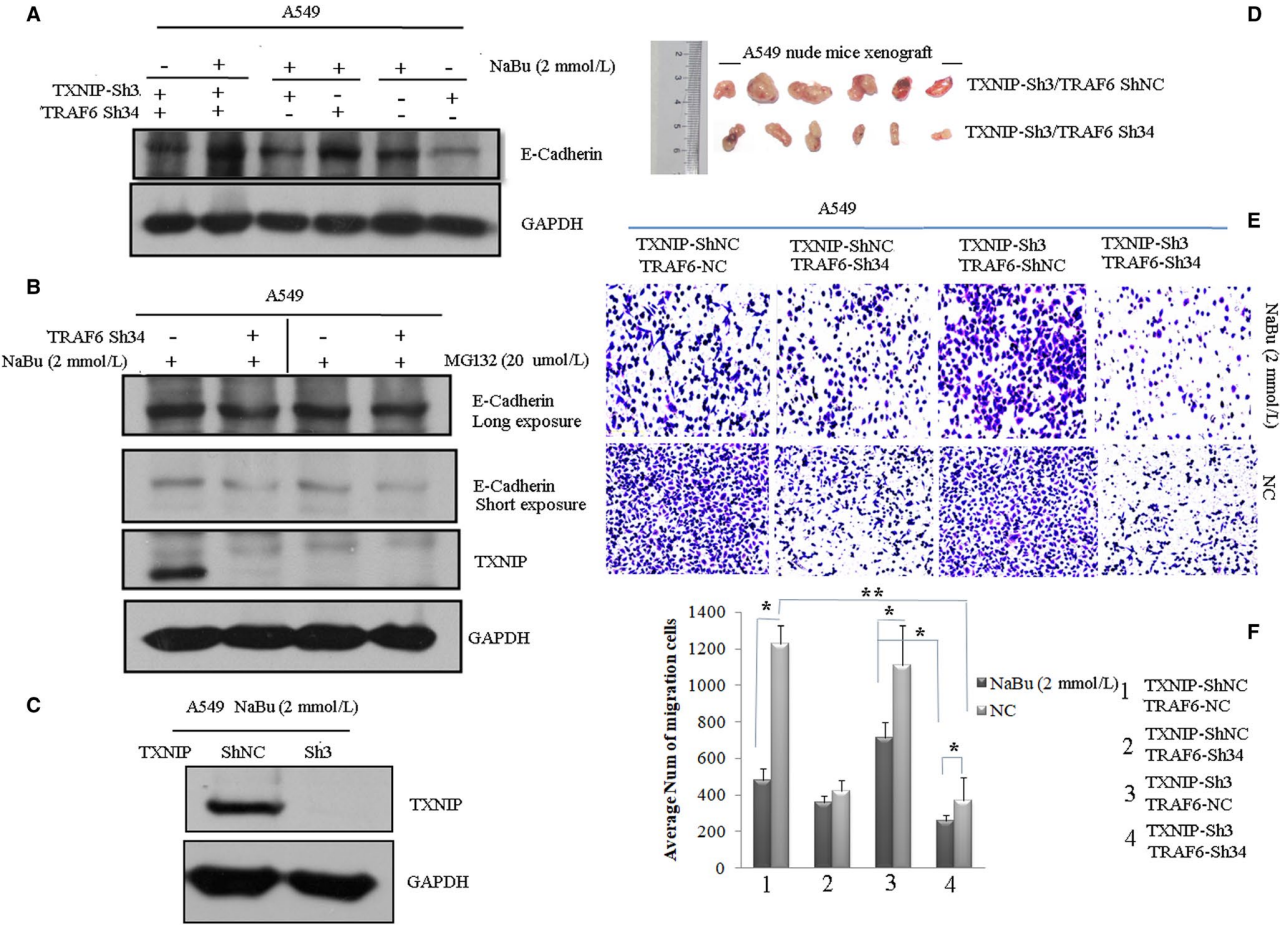
Knockdown of TRAF6 and TXNIP expression in A549 cells affects its proliferation and migration in a NaBu‐dependent manner. A, A549 cell expressing TXNIP and TRAF6 single or double shRNA (sh34 for TRAF6 and sh3 for TXNIP) were treated with or without 2 mmol/L NaBu for 24 h, cell lysates were subjected to western blot with anti‐E‐Cadherin antibody. B, A549 cell stably expressing TRAF6 shRNA(sh34) or scramble shRNA were treated with 2 mmol/L NaBu for 24 h and 20 µmol/L MG132 for 12 h, respectively. Cell lysates were subjected to western blot with anti‐E‐Cadherin and anti‐TXNIP antibodies. C, The assessment of TXNIP shRNA (sh3) knockdown efficiency was confirmed by immunoblotting with anti‐TXNIP antibody in context of 2 mmol/L NaBu treatment for 24 h. D, Photographs of tumors excised from model nude mice. Nude mice xenograft experiments were performed with the A549 cells stably expressing TXNIP shRNA(sh3) and TRAF6 scramble shRNA(NC), or expressing shRNAs for both TRAF6 and TXNIP(sh3 for TXNIP, sh34 for TRAF6) and injected into the nude mice with a dose of 10^7^ cells per mouse. E, Cell migration assay was performed with A549 cells expressing TRAF6 and TXNIP single or double shRNAs knockdown, respectively, with or without NaBu treatment. The migrated cells were detected by crystal violet staining. F, Statistical analysis was performed for the average number of migrated cells from three independent experiments and results were shown as the mean values (±SD). Student‐*t* test was used for the *P* value assay. *P < .05,**P < .01

To determine whether TXNIP‐TRAF6 signal modulates the proliferation and migration of A549 cells, we used cell lines stably expressing shRNA for TXNIP or TRAF6 or both TXNIP and TRAF6. Firstly, we checked the expression of E‐cadherin in NaBu‐treated cells. The levels of E‐cadherin expression in each cell lines were as follows (from high to low): TRAF6 sh34/TXNIP sh4 cells, TRAF6 sh34 cells, wild‐type cells and TXNIP sh4 cells, in response to NaBu treatment (Figure 6A). Next, we tested the expression of E‐cadherin in TRAF6 sh34knockdown cells treated with or without combination of NaBu and/or MG132. The results showed the expression of E‐cadherin was slightly reduced in TRAF6sh34 cells treated with both NaBu and MG132 as compared to the wild‐type cells (Figure 6B). We also tested the cell proliferation via nude mice xenograft experiment, we observed nude mice injected the cells expressing TXNIP sh4 had larger tumor size than that of cells expressing TXNIP sh4/TRAF6 sh34 (Figure 6D). The endogenous knockdown efficiency of TXNIP sh4 was confirmed by western blotting in Figure 6C. At last, we tested the cell migration in response to NaBu stimuli. Four kinds of cell lines, TRAF6 sh34, TXNIP sh4, and TRAF6 sh34/TXNIP sh4 cell lines and negative control(NC) cells were included into this assay. Among all tested cell lines, the least number of migrated cells was TXNIP sh4/TRAF6 sh4 cells, but the most TXNIP sh4 cells when treated with 2 mmol/L NaBu for 24 hours (Figure 6E, upper pane.; However, in the vehicle‐treated group, the least number of migrated cells was TXNIPsh4/TRAF6 sh34 cells and most wild‐type cells(Figure 6E, low panel). The statistical analysis for three independent experiments of Figure 6E is showns in Figure 6F.

## 4 DISCUSSION

Recently, TRAF6 was confirmed to be an important oncogene and a constitutive NF‐kB activator in RAS‐driven lung cancers.^4^ Previously, we showed NaBu could induce A549 cell death and TXNIP expression.^15^, ^16^ Actually, TXNIP is a tumor suppressive gene and induces cancer cell death.^11^ Here, we found TRAF6 could regulate NaBu‐mediated TXNIP gene expression (Figures 2 and 3). Another interesting discovery was that TRAF6 interacted with and polyubiquitylated TXNIP in A549 cells (Figure 4). Currently, TXNIP polyubiquitylation modification was only reported by ITCH E3 ligase, and ITCH accelerated TXNIP degradation after its polyunbiquitylation.^11^, ^19^ Here, we showed that TXNIP interacted with TRAF6 with the PPxY motif, which is also responsible for the interaction with ITCH.^19^ Moreover, TRAF6 stabilizes TXNIP protein but ITCH promoted degradation of TXNIP protein via polyubiquitylation. These data indicate TRAF6 and ITCH might compete with each other to interact with TXNIP and then maintain the level of TXNIP protein in cells. This deduction should be further confirmed in future. As an oncogene, TRAF6 through regulating TXNIP expression and protein stability, thus brakes the tumorgenesis of NSCLC. Confusingly, we also observed TXNIP mutant with C‐terminus 100‐aa deletion displays a high level of polyubiquitylation (data not shown). A possible explanation is that there might be an undiscovered E3 ligase‐mediating TXNIP polyubiquitylation, which is not dependent on the interaction of PPxY motif in TXNIP.

Enhanced TXNIP expression induced pro‐capase‐3, but not pro‐caspase‐1 activation (Figure 5). Previously reports showed TXNIP could activate pro‐caspase‐1 and mediate inflammation through NLRP3.^12^ However, in our current observation, the enhanced expression of TXNIP protein promoted the activation of pro‐caspase‐3 and the expression of Bax, but not the activation of pro‐caspase‐1, in response to NaBu treatment. Those results indicate TXNIP performs its functions dependent on the environmental stimuli. We also found knocking down both TXNIP and TRAF6 genes diminished proliferation and migration of A549 cells. These results were actually contradictory to the role of TRAF6, which could promote lung cancer development. Of course, we cannot exclude the possibility of other signaling pathways involved in the process. So in our coming investigation, we will scrutinize the inherent molecular mechanism.

Taken together, we revealed a novel TRAF6‐TXNIP signal in A549 cells, which could be regulated by NaBu treatment. Through this signalling pathway, the tumorigenic ability of TRAF6 can be alleviated by crosstalking a tumor suppressive gene TXNIP, leading to a decline in tumogenesis.

## DISCLOSURE STATEMENT

The authors have no conflicts of interest to declare.

## AUTHOR CONTRIBUTIONS

XXQ performed and designed the experiments, prepared the manuscript, and contributed to data correction; XYX performed some experiments. CHY reviewed the manuscript.

## Data Availability

The data used to support the findings of this study are available from the corresponding author upon request.
